# StimVision: smartphone video kinematics to optimize DBS programming in Parkinson’s disease

**DOI:** 10.1038/s41531-026-01335-6

**Published:** 2026-04-20

**Authors:** Florian Lange, Philipp Köberle, Gamze Adaçay, Diego L. Guarin, Jens Volkmann, Robert Peach, Martin M. Reich

**Affiliations:** 1 Department of Neurology, University of Würzburg, Würzburg, Germany; 2 Movement Estimation and Analysis Laboratory, Department of Applied Physiology and Kinesiology, University of Florida, Gainesville, FL USA

**Keywords:** Computational biology and bioinformatics, Neurology, Neuroscience

## Abstract

Deep brain stimulation (DBS) improves motor function in Parkinson’s disease, yet programming remains labor-intensive and largely subjective. We evaluated a smartphone video-based kinematic framework (StimVision) for objective, within-session optimization of DBS settings and characterization of therapeutic motor signatures. Fifteen patients with subthalamic DBS performed repetitive hand opening–closing while multiple stimulation programs were tested in the medication-off state. Markerless pose estimation from 60 fps smartphone video generated 23 quantitative kinematic features. A patient-specific Dynamically Weighted Improvement Score (DWIS) ranked programs by composite improvement relative to DBS-off. The framework identified a unique optimal program for each patient, with robust ranking stability. Group-level improvements at the optimal setting were dominated by gains in speed and rhythm metrics, including mean velocity, closing speed, and movement frequency, alongside reduced intra-sequence decay. Sparse principal component analysis revealed three kinematic domains—Movement Speed, Movement Consistency, and Rhythm & Timing. Structural comparison with a levodopa cohort demonstrated substantial overlap in speed and consistency domains but divergence in timing-related features. Smartphone-based kinematics enable objective DBS optimization and provide a shared quantitative framework for comparing electrical and pharmacological therapies.

## Introduction

Deep brain stimulation (DBS) is one of the most effective therapies for motor symptoms in Parkinson’s disease (PD). Yet, despite decades of clinical experience, its therapeutic potential is still constrained by how stimulation parameters are selected. Programming decisions are typically based on observer-rated motor scales and iterative trial-and-error adjustments, an approach that scales poorly with the expanding parameter space of modern DBS systems and remains inherently subjective at the point of care^1,2^. As a result, clinicians lack a quantitative, reproducible metric that captures acute motor response and allows candidate programs to be compared within a single session.

Computer vision provides a promising solution to this limitation. Markerless pose estimation applied to standard smartphone video can extract sensitive kinematic signatures of motor performance without specialized hardware. Using our previously validated open-source platform^3^, video-based kinematics have been shown to detect subtle motor deficits and treatment-induced changes in movement disorders, complementing earlier objective programming approaches that relied on wearable sensors, invasive recordings, or dedicated laboratory equipment^4–7^. Beyond single metrics, recent large-scale levodopa studies further suggest that motor improvement can be decomposed into stable, interpretable kinematic domains, providing a quantitative vocabulary for therapeutic effects across patients and interventions^7^.

Here, we conceptualize DBS programming as a quantitative selection problem. Using standardized 60 fps smartphone videos of repetitive hand opening–closing, we extract 23 markerless kinematic features and compute a patient-specific Dynamically Weighted Improvement Score to rank stimulation programs and estimate each patient’s maximal acute DBS response within a single programming session. We further summarize multivariate kinematic changes into interpretable domains and examine their structural correspondence with levodopa-induced improvement in a previously characterized cohort. This framework establishes a device-agnostic, objective approach to DBS optimization and provides a common kinematic language for comparing electrical and pharmacological therapies in Parkinson’s disease.

## Results

### Patient-level feasibility and optimization

**Fig. 1:**
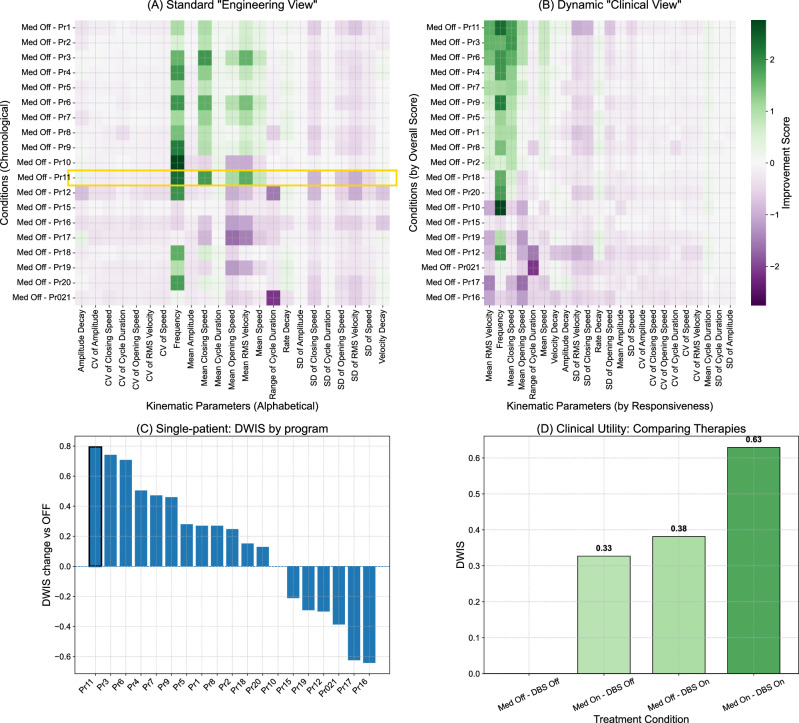
Clinical Applications of the StimVision Analysis Framework for Individual Patients. **A** *“Engineering View”*. Heatmap of improvement relative to baseline, with stimulation programs ordered chronologically by program number on the y-axis. The algorithmically selected best program (e.g., Med Off – Pr11) is highlighted. **B** *Dynamic “Clinical View”*. The same matrix, re-ordered for bedside interpretation: columns (kinematic parameters) are sorted by this patient’s responsiveness, and rows (conditions/programs) by the composite Dynamically Weighted Improvement Score (DWIS; higher = better). Reordering reveals a coherent block of improvement in the top-left and lifts the most effective programs to the top, together with the patient’s kinematic response signature. **C** *Single-patient waterfall: programs vs OFF*. Bar plot of ΔDWIS = DWIS(program) − DWIS(OFF) for every tested program in this session, sorted best to worst and anchored at zero by the OFF condition. **D** *Clinical Utility: Comparing therapies*. Summary bar chart for an exemplary patient across therapy states (Medication Off/On × DBS Off/On), recorded opportunistically outside the main medication-off protocol to illustrate the broader contextualizing potential of the framework. DWIS condenses multi-parameter kinematics into a single metric to compare therapeutic efficacy at a glance; in this illustration, combination therapy (Med-on and DBS-on) shows the largest composite improvement.

The software successfully generated a clear per-patient ranking and identified a single optimal DBS-on program for all 15 participants. The selection of this optimal program was stable; our sensitivity analysis confirmed that the top-ranked program remained unchanged across a wide range of shrinkage parameters (*λ* = 0–0.45) for 13/15 in the cohort (Supplementary Fig. S1). This demonstrates that the findings are stable and not an artifact of the specific λ value (0.1) chosen for the main analysis. To illustrate how these outputs surface at the bedside, Fig. 1 shows the per-patient decision display for a representative patient session: an “engineering view” of raw per-program improvements (Fig. 1A), a clinician-oriented re-ordering by the Dynamically Weighted Improvement Score (DWIS) that lifts the best programs and their response signature (Fig. 1B), a ΔDWIS waterfall anchored at OFF summarizing separations between programs (Fig. 1C), and an example therapy comparison (Fig. 1D).

The waterfall plot for the representative patient (Fig. 1C) suggested that more programs improved function than worsened it. To quantify this trend at the group level, we aggregated the DWIS for all tested programs across all 15 participants. Across the 150 unique patient-program combinations, a substantial majority (126/150, 84.0%) resulted in a positive DWIS (improvement), whereas only 24 (16.0%) resulted in a negative DWIS (worsening), a ratio of approximately 5.2 to 1. This trend was consistent at the individual level, where a median of 88.9% (IQR: 70.8–100.0%) of programs tested per-patient led to an improvement over their DBS-off baseline. Thus, the DWIS framework objectively quantified a wide therapeutic window, identifying a clear majority of beneficial programs while also discriminating the smaller subset of settings that were detrimental relative to baseline. To quantify whether an objectively identifiable “best” program existed within patients, we computed the separation between the top-ranked and second-ranked programs (ΔDWIS_top2). Across patients, the top-ranked program was consistently separated from the second-ranked program (median ΔDWIS_top2 = 0.12, Wilcoxon signed-rank test vs 0, *p* < 0.001). Cohort-level rank-ordered ΔDWIS curves further demonstrated a structured and monotonic decline in program scores rather than a flat distribution (Supplementary Fig. S2).

### Group-level kinematic improvements at the optimal DBS setting

**Fig. 2:**
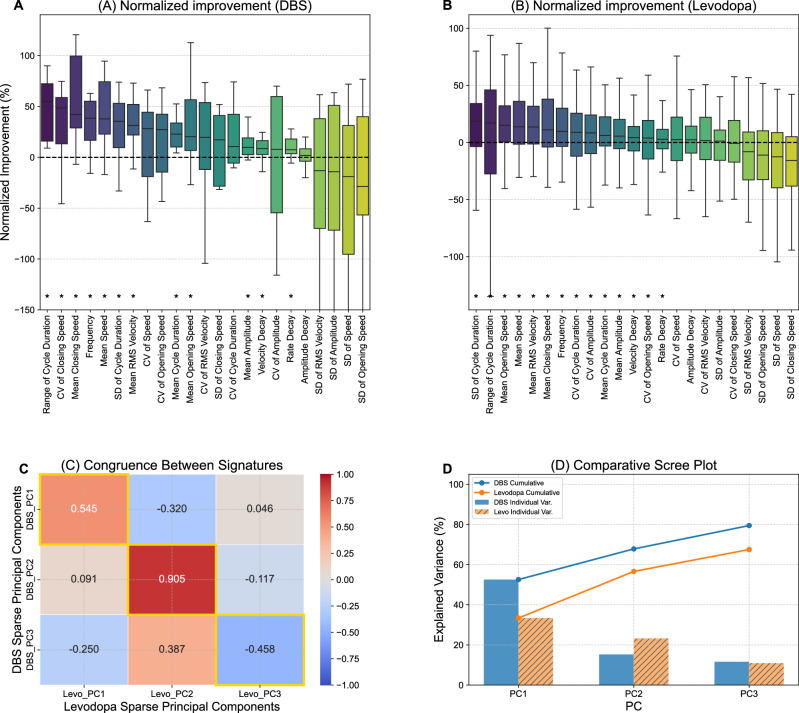
Comparison of Normalized Therapeutic Effects and Distilled Kinematic Signatures for DBS and Levodopa. **A**, **B** Boxplots show the normalized percent change for each kinematic parameter relative to the DBS-off baseline (positive = improvement after orientation; Methods). For DBS (**A**), values are taken at each patient’s session-optimal setting; for levodopa (**B**), values are from a previously analyzed medication-on cohort. Parameters on the x-axis are ordered by their median improvement in the DBS panel (**A**); the same order is applied to levodopa (**B**). Each box shows the IQR with median line; whiskers = 1.5 × IQR. Asterisks identify parameters with a median improvement significantly >0 after BH-FDR correction across 23 tests (DBS: 12 significant; levodopa: 14 significant). Because the cohorts and protocols differ, between-panel magnitudes are not directly comparable. **C** The congruence matrix compares the kinematic signatures extracted via sparse PCA (sPCA) for DBS and levodopa. Each axis represents the three principal components retained for each treatment (DBS: y-axis; levodopa: x-axis). Cell values show Tucker’s congruence coefficient (*Φ*), where values approaching +1 (red) indicate high structural similarity and values near 0 (white/gray) indicate no similarity. Gold borders highlight the diagonal, i.e., pairs of components interpreted as representing the same kinematic domain. The three domains are labeled PC1: Movement Speed, PC2: Movement Consistency, and PC3: Rhythm & Timing, based on the dominant feature loadings of each component. High congruence on the diagonal for PC2 (*Φ* = 0.905) indicates that the consistency signature of DBS and levodopa are nearly identical. PC1 shows moderate congruence (*Φ* = 0.545), and PC3 shows an inverse relationship (*Φ* = −0.458), suggesting that while the timing domain captures similar features, its directional expression differs between the two therapies. **D** The comparative scree plot visualizes the proportion of explained variance for each of the three retained components. Bars show individual component variance for DBS (blue, solid) and levodopa (orange, hatched); lines show cumulative explained variance. The three components together account for 80.3% of total variance for DBS and 67.5% for levodopa.

Across 23 parameters, 14 remained significant after BH-FDR correction (Supplementary Table S2). The five parameters with the largest effect sizes were Mean RMS Velocity (median +31.3%; *q* = 1.7 × 10^−^⁹), Mean Closing Speed ( + 42.2%; *q* = 2.0 × 10^−^⁶), Mean Speed ( + 37.8%; *q* = 1.2 × 10^−^⁸), Frequency ( + 38.4%; *q* = 2.0 × 10^−^⁶), and Frequency Decay (reduction in intra-sequence slowing; −7.5%; *q* = 0.022) (Fig. 2).Full results for all 23 parameters are reported in Supplementary Table S1.

To verify robustness of these findings and to address potential bias introduced by percent normalization, we re-analyzed all 23 parameters using linear mixed-effects models on raw values (fixed effect: condition; random intercept: subject). The parameters identified in the nonparametric analysis remained significant after FDR correction (all *q* < 0.01; Supplementary Table S2), confirming the stability of the results across statistical frameworks.

To summarize multivariate effects, Sparse PCA (pre-specified, 3 components) identified three interpretable kinematic domains (Fig. 2). In an exploratory comparison to a published levodopa cohort (Lange et al., 2025), domain structures showed high congruence for Movement Consistency (*Φ* = 0.905) and moderate congruence for Movement Speed (*Φ* = 0.545), with an inverse structural relationship for Rhythm & Timing (*Φ* = −0.458), suggesting that while both therapies modulate timing-related features, their directional expression differs. A global permutation test of overall structural similarity was not significant (*P* = 0.093), indicating similar but nonetheless structurally distinct treatment effects. These cross-therapy findings address domain structure only and do not compare effect size magnitudes between therapies.

### Clinical validation of ΔDWIS

To assess convergent validity with clinician-rated motor performance, we analyzed the association between ΔDWIS and MDS-UPDRS item 3.5 improvement across all tested programs. In a linear mixed-effects model with random intercepts for patient-hand, ΔDWIS significantly predicted clinical motor improvement (*β* = 0.70, SE = 0.15, *z* = 4.63, *P* < 0.001; 95% CI 0.40–1.00), indicating strong agreement between the kinematic ranking metric and clinician-rated response.

To address potential bias introduced by baseline normalization, we additionally fitted a mixed-effects model on raw UPDRS scores including baseline severity as a covariate (Score ~ ΔDWIS + BaselineScore). In this model, ΔDWIS remained an independent predictor of lower (i.e., better) UPDRS scores (*β* = −0.67, SE = 0.15, *z* = − 4.48, *P* < 0.001; 95% CI −0.97 to −0.38), whereas baseline severity was not significant (*P* = 0.30). These findings confirm that the association between ΔDWIS and clinical motor performance is robust and not driven by baseline scaling effects.

## Discussion

This study establishes a quantitative framework for objective, patient-specific optimization of deep brain stimulation (DBS) in Parkinson’s disease. By using smartphone-based computer vision, we demonstrate that acute motor response to DBS can be captured reproducibly, ranked within a single programming session, and summarized along physiologically interpretable kinematic domains. Rather than relying on observer-rated scales or heuristic parameter selection, this approach provides a data-driven representation of therapeutic response that is both clinically actionable and mechanistically informative.

At the individual level, our framework consistently identified a session-optimal stimulation setting for every patient and demonstrated robust ranking stability across sensitivity analyses. At the group level, DBS-induced improvement was dominated by gains in movement speed and consistency, with several features remaining significant after correction for multiple testing. Importantly, dimensionality reduction revealed that these multivariate changes converge onto a small number of stable kinematic domains, suggesting that acute DBS effects can be meaningfully described within a low-dimensional motor control space.

This domain-based representation enabled a direct structural comparison between DBS- and levodopa-induced motor improvement. We observed a pronounced overlap in domains related to movement speed and consistency, alongside divergence in rhythm and timing-related features. These findings argue against a simplistic view of DBS as merely “electrical levodopa” and instead support a model in which electrical and pharmacological therapies partially converge on shared motor control mechanisms while exerting distinct effects on specific aspects of movement organization.

Personalizing DBS programming remains a central clinical challenge, particularly as modern multi-contact electrodes expand the stimulation parameter space beyond what can be navigated efficiently by trial-and-error approaches^8^. Objective, biomarker-guided strategies have therefore gained increasing attention. Early work using wearable sensors demonstrated that kinematic tuning maps can support data-driven parameter selection^9,10^. Our framework builds directly on these concepts while removing the need for dedicated hardware.

By combining standard smartphone video with our open-source VisionMD platform^3^, we extract high-resolution kinematic profiles using ubiquitous, low-cost technology. This substantially lowers barriers to clinical adoption and enables scalability to routine care and remote settings. The resulting “Dynamic Clinical View” translates complex multivariate data into an intuitive ranking of stimulation programs, while preserving transparency regarding which motor domains drive improvement in a given patient.

Beyond its clinical utility, this framework provides a quantitative vocabulary for interrogating the mechanisms of DBS. Sparse principal component analysis distilled DBS-induced motor improvement into distinct, physiologically interpretable domains. The strong congruence between DBS and levodopa in domains related to movement speed and consistency is consistent with a shared capacity of both therapies to suppress pathological beta-band synchronization within cortico-basal ganglia circuits^11^.

At the same time, the reduced congruence observed for rhythm and timing-related domains suggests mechanistic divergence. This distinction aligns with electrophysiological and EMG studies demonstrating that DBS and dopaminergic therapy differentially modulate temporal aspects of motor output, including burst structure and movement regularity^12^. Together, these findings support a model in which DBS and levodopa converge on core motor activation pathways while exerting partially distinct effects on the temporal organization of movement.

While this study provides a robust proof-of-concept, several limitations should be acknowledged when interpreting the findings, and these directly inform future directions.

First, the cohort size (*n* = 15) is modest. Second, the comparative analysis with levodopa relied on a separate, historical cohort, which, while powerful, introduces potential confounding variables that could influence the comparison. Therefore, validation in larger, prospective cohorts using a single, standardized protocol is a critical next step. An ideal future study would employ a within-subject design, directly comparing the kinematic signatures of DBS and levodopa within the same patients to provide the most definitive analysis of their shared and divergent mechanisms.

Third, our current optimization algorithm focuses solely on maximizing therapeutic motor benefit from a single task. A truly optimal clinical setting, however, represents a trade-off between efficacy and tolerability. Our framework does not yet incorporate the systematic quantification of stimulation-induced side effects (e.g., dysarthria, paresthesia). Future iterations should aim to integrate objective or patient-reported measures of side effects, creating a multi-objective optimization algorithm that can recommend a setting that best balances motor improvement with patient comfort.

Fourth, the analysis was based exclusively on the repetitive hand opening–closing task. While this is a highly sensitive measure of appendicular motor control, the kinematic domains identified may not fully generalize to other critical motor symptoms. Future work should expand this framework to include a broader battery of motor tasks to assess effects on gait, balance, handwriting, and other axial or complex motor functions, providing a more holistic view of the therapeutic impact.

Finally, tremor-dominant patients were excluded from the present cohort, as the hand-aperture signal is not robust to oscillatory tremor displacement. Validation of the framework in tremor-dominant patients, potentially incorporating tremor-specific signal decomposition or alternative kinematic features, remains an important avenue for future work.

A prospective study directly comparing StimVision-guided programming with independently clinician-optimized stimulation settings will be necessary to determine whether kinematic optimization improves or accelerates standard clinical programming.

## Methods

### Study design, participants, and setting

This was an observational proof-of-concept study conducted at a single academic center. The protocol complied with the Declaration of Helsinki and received approval from the local ethics committee (Ethik-Kommission an der Medizinischen Fakultät der Julius-Maximilians-Universität Würzburg; Ethics approval number: 100/24-am). Written informed consent was obtained from all participants prior to any procedures.

We studied 15 individuals with idiopathic Parkinson’s disease (PD) treated with subthalamic nucleus deep brain stimulation (DBS). Inclusion criteria were idiopathic PD with a documented levodopa response, implanted DBS for ≥6 months, and ability to complete the motor task in the medication-off state. Exclusion criteria included atypical parkinsonism, major neurological or psychiatric comorbidity, or cognitive impairment precluding consent. Patients with tremor-dominant disease phenotype were excluded, as oscillatory hand landmark displacement would contaminate the hand-aperture signal and confound bradykinesia-sensitive kinematic features. In the examined hands, MDS-UPDRS-III tremor subscores (items 3.15–3.18) were 0 in all participants in the medication-off state, indicating absence of clinically detectable tremor during testing. Candidate stimulation programs were evaluated in the medication-off state as part of routine clinical programming. Programs reflected either a clinician-guided selection. Frequency and pulse width were held constant within each session; bipolar configurations were not used, and directional stimulation was applied only when clinically indicated. Recordings were obtained after an approximately 2-min wash-in period. Clinicians were blinded to DWIS outputs during programming. Baseline characteristics are summarized in Table 1.

**Table 1:** Summary of patient demographics and clinical characteristics

Characteristic	Value (*n* = 15)
Age (years), mean ± SD	64.6 ± 9.3
Sex, *n* (%)	
Male	11 (73.3%)
Female	4 (26.6%)
Disease Duration (years), mean ± SD	10.6 ± 3.2
Study Protocol, *n* (%)	
Systematic Monopolar Review	10 (66.6%)
Routine Clinical Programming	5 (33.3%)
Levodopa Equivalent Daily Dose (LEDD), mg/day, mean ± SD (*n* = 15)^a^	441.2 ± 219.1

This table presents the summary statistics for the 15 participants included in the study (full breakdown in Table S1). Continuous variables (Age, Disease Duration) are presented as mean ± standard deviation (SD). Categorical variables (Sex, Study Protocol) are presented as counts (*n*) and percentages (%). LEDD denotes the Levodopa Equivalent Daily Dose.

^a^One patient was not receiving dopaminergic therapy at the time of the study and was therefore excluded from the LEDD calculation.

### Terminology and Framework

We here distinguish between two components: (i) VisionMD, our open-source computer vision platform that extracts kinematic features from raw smartphone video^3^, and (ii) StimVision, our analysis and decision-support layer that applies these features to rank stimulation programs. Throughout the manuscript, we refer to this combined process as the StimVision framework.

### Motor task, video acquisition, and feature extraction

All recordings were performed after overnight withdrawal of dopaminergic medication ( ≥ 12 h; medication-off). The task was repetitive hand opening–closing (MDS-UPDRS item 3.5). Videos were captured on a standard smartphone (60 fps; 1920×1080), following the standardized MDS-UPDRS item 3.5 recording protocol (approximately 10–15 s per trial). Kinematic analyses and DWIS computations were performed separately for each hand; the hand contralateral to the hemisphere being programmed was used to evaluate that hemisphere’s stimulation settings.

Videos were processed with the open-source and previously validated VisionMD platform^3^ using MediaPipe-based markerless 2D hand pose estimation^13^. A one-dimensional hand-aperture signal was derived as the Euclidean distance between the third-digit tip and wrist landmark, normalized by hand length. From this signal, 23 kinematic parameters were computed to quantify amplitude, speed/vigor, temporal rhythm, variability, and decrement (e.g., amplitude or velocity decay). We treated programming as a within-patient selection problem. For each patient, the primary estimand was the maximal acute DBS response achievable among clinically selectable programs during a single session, defined as the difference between that patient’s optimal DBS-on condition and the DBS-off baseline for each kinematic parameter.

### Per‑patient ranking via dynamic weighting

**Fig. 3:**
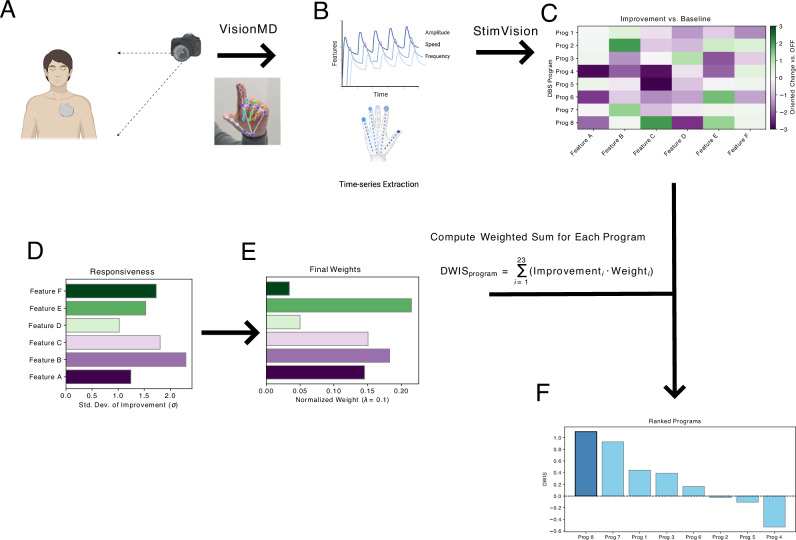
The StimVision Computational Framework for Objective DBS Program Optimization. This figure illustrates the step-by-step workflow for calculating the Dynamically Weighted Improvement Score (DWIS) to identify the optimal program. **A** A patient performs a standardized motor task while being recorded with a smartphone video camera. Raw video is processed in *VisionMD*, which performs markerless 2D hand pose estimation. **B** Kinematic time-series features (e.g., amplitude, speed, frequency) are extracted by *VisionMD*. **C** For each tested DBS program, *StimVision* calculates improvement for each feature relative to the patient-specific DBS-off baseline, forming an improvement matrix where each row represents a program’s kinematic signature. **D** *StimVision* then quantifies feature “responsiveness” as the standard deviation of improvement across programs. **E** Responsiveness scores are normalized to create patient-specific weights (optional shrinkage tested in robustness analysis). **F** The weighted improvements are summed into a DWIS for each program, producing an objectively ranked list of all tested programs and identifying the patient’s optimal program for the session. Created with BioRender.com.

To identify the optimal DBS program for each patient, we developed a computational framework that transforms raw video kinematics into a ranked list of programs via a patient-specific metric, the Dynamically Weighted Improvement Score (DWIS). The complete end-to-end workflow, from video acquisition to the final ranked output, is illustrated in Fig. 3. The following steps detail this process.

We first calculated the raw change for each of the 23 kinematic parameters relative to the DBS-off baseline and oriented each change so that larger values always reflected clinical improvement (e.g., multiplying “lower-is-better” variability metrics by −1). To quantify which parameters discriminated between DBS programs within a given patient, we defined a responsiveness index for each parameter as the standard deviation of its oriented improvement values across all DBS-on settings. This responsiveness measure captures the extent to which a feature varies across candidate programs within that session.

Dynamic weights were then obtained by normalizing these responsiveness values to sum to one across parameters. For each setting, the DWIS was computed as the weighted sum of the 23 oriented improvements. The setting with the highest DWIS was defined as that patient’s optimal program for the session and was used in group-level analyses.

As a robustness analysis, we additionally evaluated a generalized shrinkage formulation that blends responsiveness-derived weights with uniform weights across responsive parameters (λ ∈ [0, 0.45]). Rankings were stable across this range in the majority of patients (Supplementary Fig. S1), indicating that program selection was not dependent on a specific regularization choice.

### Group‑level statistical analysis

Within‑patient optimal‑vs‑baseline differences were aggregated across patients. For each parameter we report the median percent change (with IQR) and tested whether the median improvement exceeded zero using the one‑sample Wilcoxon signed‑rank test, applying Benjamini–Hochberg-FDR correction across 23 parameters. To emphasize estimation, 95% confidence intervals for medians were obtained via nonparametric bootstrap (10,000 resamples). All analyses used Python 3.10 (pandas, numpy, scipy, scikit‑learn, matplotlib). To quantify within-patient-program separability, we additionally computed, for each patient, the difference in DWIS between the top-ranked and second-ranked program (ΔDWIS_top2) and between the top-ranked and median program. These separation metrics were aggregated across patients and tested against zero using one-sample Wilcoxon signed-rank tests. For visualization, we generated cohort-level rank-ordered ΔDWIS curves (best → worst) across all tested programs per patient (Supplementary Fig. 1).

### Clinical concordance analysis

To evaluate convergent validity with clinician-rated motor performance, we analyzed the association between ΔDWIS and MDS-UPDRS item 3.5 improvement across all tested programs. For each patient-hand and program, UPDRS improvement was defined as the baseline (medication-off / stimulation-off) score minus the program-specific score. We fitted a linear mixed-effects model with UPDRS improvement as the dependent variable, ΔDWIS as a fixed effect, and a random intercept for patient-hand to account for within-subject clustering of multiple programs. Model parameters were estimated using restricted maximum likelihood. In addition, we computed per-patient Spearman rank correlations between DWIS-based and UPDRS-based program rankings and quantified agreement between the clinically optimal and DWIS-optimal program selections.

### Exploratory domain analysis and cross‑therapy structure comparison

To summarize multivariate effects into interpretable domains, we applied sparse Principal Component Analysis (sPCA) to the matrix of DBS improvement scores. Hyperparameters were fixed a priori by adopting the settings from our prior levodopa study (n_components = 3, alpha = 1; random_state = 0) and were not tuned on the DBS data. Component-loading 95% CIs were obtained via patient-level bootstrap (10,000 resamples). For context, we repeated the same sPCA procedure on a previously analyzed levodopa cohort (*n* = 154), restricted to the overlapping feature set, then quantified cross-therapy structural similarity using Tucker’s congruence (*Φ*) and a 10,000-iteration permutation test for global similarity. These analyses target pattern structure of component loadings; they do not compare between-therapy effect-size magnitudes.

## Supplementary information

Supplementary material.

## Data Availability

The derived kinematic datasets and analysis matrices generated during the current study are publicly available at: https://github.com/Flolan2/StimVision_data.

## Code availability

The core VisionMD computer vision pipeline used for feature extraction is open-source and available at https://www.visionmd.ai/. The StimVision analysis pipeline used in this study is publicly available at https://github.com/Flolan2/StimVision_data.
