# Supplementary material for: StimVision: smartphone video kinematics to optimize DBS programming in Parkinson’s disease
Source: NPJ Parkinsons Dis. 2026 Apr 20;12:100. doi: 10.1038/s41531-026-01335-6 (PMC13096112; doi:10.1038/s41531-026-01335-6)
Supplement: Supplementary file 1 — Supplementary material. [file 41531_2026_1335_MOESM1_ESM.docx]

# Supplementary material

| *Patient ID* | *Age/Sex* | *Disease Duration (years)* | *Levodopa Equivalent Daily Dose (LEDD, mg/day)* | *MDS-UPDRS III (Med-OFF/STIM-OFF)* |
| --- | --- | --- | --- | --- |
| *01* | *57/F* | *18* | *425* | *49* |
| *02* | *77/M* | *9* | *340* | *25* |
| *03* | *65/M* | *10* | *300* | *41* |
| *04* | *54/M* | *12* | *302* | *54* |
| *05* | *70/M* | *14* | *915* | *64* |
| *06* | *69/F* | *9* | *475* | *32* |
| *07* | *64/M* | *8* | *300* | *46* |
| *08* | *76/M* | *12* | *480* | *39* |
| *09* | *54/M* | *8* | *150* | *60* |
| *10* | *43/M* | *6* | *474* | *17* |
| *11* | *73/M* | *12* | *899* | *63* |
| *12* | *70/F* | *13* | *479* | *56* |
| *13* | *70/M* | *8* | *308* | *50* |
| *14* | *62/M* | *7* | *0** | *32* |
| *15* | *65/F* | *13* | *330* | *49* |

**Supplementary Table 1 | Individual Patient Demographics and Clinical Data**

This table provides detailed demographic and clinical information for each of the 15 participants included in the study. "Age/Sex" denotes the participant's age in years and sex (M=Male, F=Female) at the time of the study. "Disease Duration" was calculated from the year of diagnosis to the study year (2025). "Levodopa Equivalent Daily Dose (LEDD)" quantifies the total daily intake of dopaminergic medication.

| Kinematic Variable | Orientation | Median Change (%) | P-Value | Significant After Bonferroni Correction | 95% CI low/HIGH | Effect size (rank-biserial) |
| --- | --- | --- | --- | --- | --- | --- |
| MeanRMSVelocity | Higher | 31.3 | 0.0002 | True | 19.72 / 49.41 | 0.966667 |
| meanClosingSpeed | Higher | 42.2 | 0.0002 | True | 25.46 / 104.26 | 0.966667 |
| MeanSpeed | Higher | 37.8 | 0.0003 | True | 24.16 / 67.04 | 0.95 |
| FREQUENCYDecay | Lower | 7.5 | 0.0020 | True | 4.97 / 20.62 | 0.85 |
| Frequency | Higher | 38.4 | 0.0020 | True | 22.50 / 62.84 | 0.85 |
| velocityDecay | Lower | 8.7 | 0.0054 | False | 2.30 / 13.96 | 0.783333 |
| MeanOpeningSpeed | Higher | 20.3 | 0.0067 | False | 6.13 / 49.11 | 0.766667 |
| cvClosingSpeed | Lower | 48.5 | 0.0103 | False | 6.00 / 59.15 | 0.733333 |
| meanCycleDuration | Lower | 22.8 | 0.0125 | False | 8.75 / 42.58 | 0.716667 |
| MeanAmplitude | Higher | 8.4 | 0.0151 | False | 1.57 / 16.44 | 0.7 |
| stdCycleDuration | Lower | 35.4 | 0.0256 | False | 11.56 / 40.02 | 0.65 |
| stdOpeningSpeed | Lower | -28.6 | 0.3591 | False | -60.02 / 38.74 | -0.28333 |
| cvOpeningSpeed | Lower | 27.3 | 0.5245 | False | -11.42 / 46.88 | 0.2 |
| rangeCycleDuration | Lower | 55.0 | 0.3303 | False | 22.86 / 69.84 | 0.3 |
| StdRMSVelocity | Lower | -13.1 | 0.4543 | False | -73.76 / 37.33 | -0.23333 |
| amplitudeDecay | Lower | 1.8 | 0.4212 | False | -1.40 / 6.99 | 0.25 |
| StdSpeed | Lower | -19.0 | 0.2769 | False | -105.85 / 31.22 | -0.33333 |
| StdAmplitude | Lower | -14.1 | 0.4212 | False | -75.58 / 49.10 | -0.25 |
| cvAmplitude | Lower | 7.7 | 0.8904 | False | -55.94 / 56.77 | 0.05 |
| cvCycleDuration | Lower | 10.6 | 0.4887 | False | -10.45 / 41.89 | 0.216667 |
| cvSpeed | Lower | 28.2 | 0.4543 | False | -29.4 / 43.8 | 0.233333 |
| cvRMSVelocity | Lower | 19.6 | 0.4887 | False | -18.1 / 53.1 | 0.216667 |
| stdClosingSpeed | Lower | 17.3 | 0.7197 | False | -31.6 / 42.0 | 0.116667 |

**Supplementary Table 2 | Kinematic Improvements with DBS.**Group-level results (n=15) comparing each patient’s optimal DBS-on setting with the DBS-off baseline for 23 kinematic parameters from the hand opening–closing task. The **Orientation** column indicates the direction in which the **raw metric** is clinically better **before orientation**. Median Change (%) is shown for oriented data, where positive values uniformly indicate improvement. Statistical significance was determined by a Wilcoxon signed-rank test, with results shown before ('P-Value') and after Bonferroni correction for 23 comparisons (α ≈ 0.0022). The 95% confidence interval ('95% CI') for the median change was derived from 10,000 bootstrap resamples, and the rank-biserial correlation is provided as a standardized 'Effect Size'.

**Supplementary Figure 1 | Robustness of Optimal Program Selection to Regularization.**

This heatmap displays the optimal deep brain stimulation (DBS) program identified for each patient (y-axis) across a range of regularization parameter (λ) values (x-axis). A λ of 0 represents a fully data-driven weighting scheme, while increasing λ progressively blends these weights with a uniform distribution to reduce potential influence from measurement noise. Program labels (e.g., '1', '2', 'Off') are anonymized integer identifiers unique to each patient's set of tested conditions.

The selection of the optimal program is stable, remaining unchanged for 13 of 15 patients across a wide range of regularization strengths (λ = 0.00 to 0.40). For the two patients where the optimal program choice shifted, the change occurred only at higher levels of regularization, indicating that the top candidate programs had very similar overall benefit. This analysis confirms that the ranking method is robust and that the identified optimal programs are based on a strong therapeutic signal, not an artifact of the specific λ value (0.10) chosen for the primary analysis.


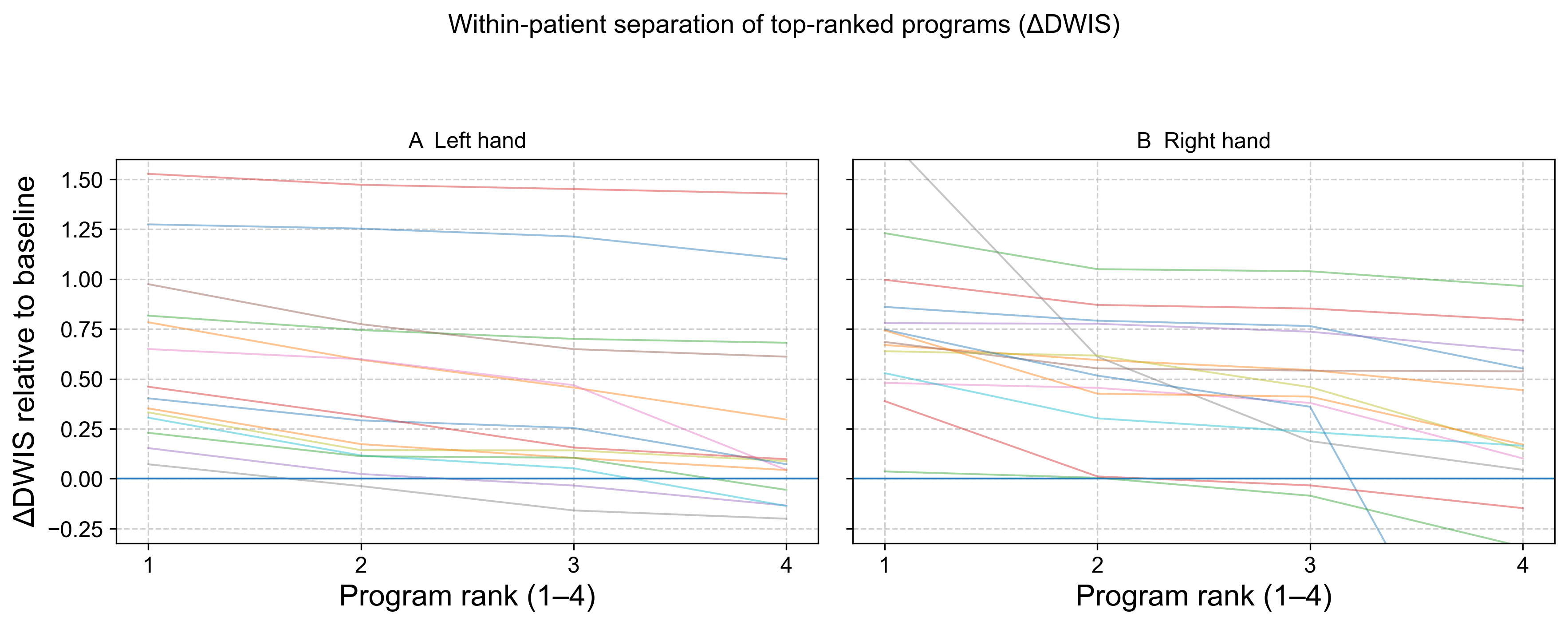


**Supplementary Figure S2.** Within-patient separation of top-ranked DBS programs. Each line represents one patient's ΔDWIS scores for their top 4 ranked stimulation programs, shown separately for left hand (A) and right hand (B). The monotonic decline in ΔDWIS across program ranks confirms a structured therapeutic window, with a clearly identifiable best program per patient, rather than a flat distribution of scores.
